# Gold Nanocluster-Encapsulated Hyperbranched Polyethyleneimine for Selective and Ratiometric Dopamine Analyses by Enhanced Self-Polymerization

**DOI:** 10.3389/fchem.2022.928607

**Published:** 2022-07-08

**Authors:** Jing Zhang, Ying Liu, Yang Liu, Wencai Liu, Fengniu Lu, Zhiqin Yuan, Chao Lu

**Affiliations:** ^1^ State Key Laboratory of Chemical Resource Engineering, College of Chemistry, Beijing University of Chemical Technology, Beijing, China; ^2^ Department of Chemistry and Chemical Engineering, Beijing Institute of Technology, Beijing, China; ^3^ Beijing Key Laboratory of Plant Resources Research and Development, Beijing Technology and Business University, Beijing, China; ^4^ Green Catalysis Center, College of Chemistry, Zhengzhou University, Zhengzhou, China

**Keywords:** hyperbranched polyethyleneimine, gold nanoclusters, dopamine analysis, self-polymerization, ratiometric fluorescence

## Abstract

The exploitation of selective and sensitive dopamine (DA) sensors is essential to more deeply understand its biological function and diagnosis of related diseases. In this study, gold nanocluster-encapsulated hyperbranched polyethyleneimine (hPEI-Au NCs) has been explored as the specific and ratiometric DA nanoprobe through hPEI-assisted DA self-polymerization reactions. The Au NCs encapsulation not only provides a fluorescent internal reference but also enhances the DA self-polymerization by weakening the proton sponge effect of the hPEI layer. Rapid and sensitive DA detection is realized through the proposed hPEI-Au NC nanoprobe with a limit of detection of 10 nM. The favorable selectivity over other possible interferents including amino acids, sugars, and salts is due to the specific self-polymerization reaction. The DA analysis in urine samples with small relative standard deviations has been accomplished with an hPEI-Au NC nanoprobe.

## Introduction

Catecholamines with specific structures can act as neurotransmitters, which are associated with neuron communication and affect brain functions (Fuxe et al., 2012). Among these catecholamines, dopamine (DA) regulates numerous biological processes and plays vital roles in the nervous, cardiovascular, and renal systems (Bucolo et al., 2019). Thus, its abnormality usually reflects the physiologic condition and is related to many diseases. For example, aprosexia related to lack of muscle control and Parkinson’s diseases are reported to be associated with DA deficiency (Kim et al., 2002). Meanwhile, energy metabolism disorder-induced Huntington’s disease is caused by the overexpression of DA (Cepeda et al., 2014). In this case, the development of sensitive and accurate analytical methods for DA quantification is significant for a deeper understanding of its biological function and early diagnosis of relevant diseases.

So far, a number of DA detection methods based on UV–vis spectroscopy, fluorimetry, Raman, electrochemical technique, and nuclear magnetic resonance imaging have been reported (Li et al., 2013; Mao et al., 2018; Ling et al., 2020; Senel et al., 2020; Ren et al., 2021). In particular, fluorescence-based DA detection approaches attract growing attention because of their low background, high sensitivity, and strong interference rejection (Qu et al., 2019; Yu et al., 2021). The commonly used mechanisms for fluorescent DA sensing can be divided into four categories: aptamer–DA binding-mediated conformation change, o-diquinone-induced fluorescence quenching, resorcinol–DA coupling-mediated formation of azamonardine, and polydopamine-regulated energy transfer (Zhang et al., 2016; He et al., 2018; Zeng et al., 2020; Ma et al., 2021). Oxidation of DA-induced production of o-diquinone is primarily applied to DA sensing with inorganic fluorophores as the references (Zhang et al., 2015; Diaz-Diestra et al., 2017). This strategy, however, is difficult to distinguish DA from other catechols. Therefore, it is appealing to explore a simple and direct strategy for selective DA perception. It is reported that hyperbranched polyethyleneimine (hPEI) can induce the spontaneous formation of polymeric DA nanoparticles with strong green fluorescence through DA self-polymerization reaction (Liu et al., 2015). This reaction is also capable to discriminate DA analogs by integrating the linear discrimination analysis technique (Sun et al., 2019). However, such a discrimination approach can only differentiate DA analogs at the µM level. Notice that ratiometric sensing systems with built-in correction characters usually display high sensitivity (Li et al., 2017; Huang et al., 2018; Yang et al., 2020a); this specific reaction, in combination with a fluorescent internal reference, may be able to construct a sensitive and selective DA sensor, which is theoretically feasible.

**FIGURE 1:**
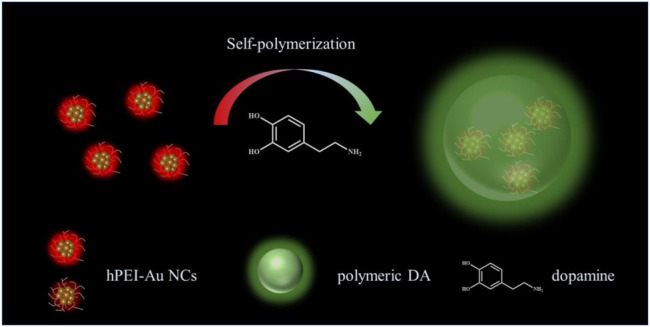
Schematic illustration of ratiometric DA detection with the hPEI-Au NC nanoprobe.

Gold nanoclusters (Au NCs) consist of several gold atoms and show unique chemical/physical properties and usually bright fluorescence (Liang et al., 2022; Liu et al., 2022). The ultrasmall size and bright emission make them potential reporters in chemo/biosensing and imaging (Chen et al., 2015; Xiao et al., 2021). Notably, the surface protecting layers largely decide the stability and application of Au NCs (Tseng et al., 2014; Lu et al., 2020). As indicated in our previous reports, hPEI with branched molecular structure and abundant amine groups can act as a good template for yielding fluorescent gold nanoclusters (Au NCs), and the emission of Au NCs is tunable by regulating hPEI/thiolate molar ratio (Yuan et al., 2017; Lu et al., 2019; Yang et al., 2020b). It is thus conjectured that Au NCs-encapsulated hPEI (hPEI-Au NCs) might endow selective and sensitive DA sensing by integrating specific reaction and ratiometric response. In this study, we tried our attempt to explore the selective and sensitive DA detection system by utilizing red-emissive hPEI-Au NCs as the reporters. The addition of DA caused an increase in green fluorescence and a decrease in red emission by DA self-polymerization–mediated formation of polymeric DA nanoparticles and alteration of the hPEI-Au NCs charge transfer pathway, which achieved selective and ratiometric DA. The schematic illustration of the DA detection mechanism using hPEI-Au NCs is shown in Figure 1. The proposed DA nanoprobes exhibited a rapid response toward DA with a limit of detection (LOD) of 10 nM (S/N = 3). The selectivity toward DA over other amino acids, small molecules, and ions was also inspected. In addition, the practical application of the proposed hPEI-Au NC nanoprobe was verified by reproducible and accurate DA analysis in urine samples.

## Methods

### Materials, Reagents, and Instruments

Chloroauric acid tetrahydrate (HAuCl_4_·4H_2_O) was purchased from damas-beta (Shanghai, China). Polyethyleneimine (25,000, branched) was purchased from Shanghai Macklin Biochemical Co., Ltd. (Shanghai, China). 11-Mercaptoundecanoic acid (MUA) was purchased from Shanghai Yuanye Bio-Technology Co., Ltd. (Shanghai, China). Dopamine (DA) and anthocyanidins (Anthos) were purchased from Aladdin Industrial Corporation (Shanghai, China). Uric acid (UA) was purchased from TCI (Shanghai, China). Urea and potassium nitrate (KNO_3_) were purchased from Xilong Scientific (Guangdong, China). Potassium chloride (KCl), sodium chloride (NaCl), and sodium sulfate (Na_2_SO_4_) were purchased from Fuchen (Tianjin, China). L-Alanine (Ala), L-lysine (Lys), and L-threonine (Thr) were purchased from Solarbio (Beijing, China). L-serine (Ser), L-arginine (Arg), and saccharose (Sac) were purchased from J&K Chemical Ltd (Beijing, China). L-ascorbic acid (AA) and catechin (Cat) were obtained from Hunan Intellijoy Biotechnology Co., Ltd. (Changsha, China). Quercetin (Que) was purchased from TCI (Shanghai, China). Anhydrous calcium chloride (CaCl_2_), glucose (Glu), hydrochloric acid (HCl), sodium hydroxide (NaOH), ethanol, disodium hydrogen phosphate (Na_2_HPO_4_), potassium dihydrogen phosphate (KH_2_PO_4_), sodium carbonate (Na_2_CO_3_), and sodium bicarbonate (NaHCO_3_) were purchased from Beijing Chemical Reagent Company (Beijing, China). All chemicals used were of the analytical-reagent grade and used without further purification. All solutions were freshly prepared with deionized water (18.2 MΩ cm, Milli-Q, Millipore, Barnstead, CA, United States).

The UV–vis absorption spectra were collected using a UV−3900H spectrophotometer (Shimadzu, Japan). Fluorescence spectra were obtained using an F-7000 fluorescence spectrophotometer (Hitachi, Japan) at a slit of 5.0 nm with a scanning rate of 2,400 nm/min. Zeta potential and hydrodynamic diameter were determined using a Malvern Zetasizer 3000HS nano-granularity analyzer (Malvern, United Kingdom). The transmission electron microscopy (TEM) images were collected using an HT7700 transmission electron microscope (HITACHI, Japan). The time-resolved fluorescence decay curve was performed on an FLS 980 (Edinburgh, United Kingdom). The pH values were measured using a benchtop pH meter (Orion plus, Thermo Fisher, United States).

### Synthesis of Red-Emissive hPEI-Au NCs

Red-emissive hPEI-Au NCs were prepared according to our previous report with slight modifications (Yuan et al., 2017). Typically, 1.1 ml hPEI (10 mM) dissolved in ultrapure water was first mixed with 50 μl HAuCl_4_ (0.1 M) to make a final solution volume of 4 ml. After 10 min stirring, 50 μl AA (0.1 M) was added. Fifteen minutes later, 100 μl MUA ethanol solution (0.1 M) was introduced into the colorless solution. The solution was stirred at room temperature for another 6 h. Finally, the resulted light yellow solution with red emission was obtained and stored at room temperature before further characterization and application.

### Sensitivity and Selectivity Measurement

First, DA stock solution (500 μM) was prepared and then diluted with ultrapure water to obtain a series of DA with a concentration gradient. To detect DA, 50 μl of DA solution with various concentrations were mixed with 50 μl of hPEI-Au NCs and 900 μl of ultrapure water to make the final volume 1 ml. After 25 min of reaction at UV light (365 nm) and 30°C, the fluorescence emission spectra were collected by an F-7000 fluorescence spectrophotometer at the excitation wavelength of 320 nm. In order to evaluate the specificity of the probe, the specificity of the metal ions, amino acids, and small molecules, including Ca^2+^, K^+^, Na^+^, Cl^−^, NO^3-^, SO_4_^2-^, urea, UA, Glu, Sac, Lys, Ala, Ser, Thr, Arg, AA, Cat, Anthos, and Que were considered. To investigate the inference, interferents were added to hPEI-Au NCs working solutions in the absence or presence of 10 μM DA. The concentrations for metal ions, small molecules, and amino acids were also 10 μM. After 25 min of reaction at UV light (365 nm) and 30°C, the fluorescence emission spectra were collected by an F-7000 fluorescence spectrophotometer at the excitation wavelength of 320 nm.

### Urine Sample Analysis

Human urine samples were obtained from a healthy volunteer who had not taken any drug/DA in the past 3 months. Urine samples were first filtered by a filter membrane with a pore size of 0.22 μm and were then ultrafiltered twice with an ultrafiltration tube with a molecular weight of 3,000 at 5,000 rpm for 8 minutes. For DA detection, 50 μl urine samples were added into a 1.5 ml centrifuge tube containing 50 μl of hPEI-Au NCs probe, and then 900 μl of ultrapure water was added to make the final volume 1 ml. After 25 min of reaction at UV light (365 nm) and 30°C, the fluorescence emission spectra were collected by an F-7000 fluorescence spectrophotometer at the excitation wavelength of 320 nm. For conducting standard addition experiments, 50 μl DA with different concentrations were firstly mixed with 50 μl of urine sample, then 50 μl hPEI-Au NCs was added immediately, and, finally, 850 μl water was added to make the final volume 1 ml. The final concentrations of added DA were 2 μM, 3 μM, and 4 μM respectively. After 25 min of reaction at UV light (365 nm) and 30°C, the fluorescence emission spectra were collected by an F-7000 fluorescence spectrophotometer at the excitation wavelength of 320 nm.

## Results and Discussion

### DA-Mediated Fluorescence Response of hPEI-Au NCs

**FIGURE 2:**
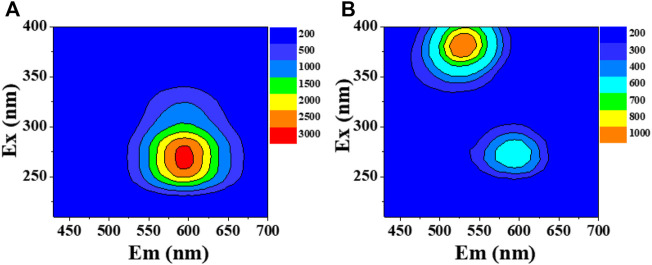
3D fluorescence emission spectra of hPEI-Au NCs in the absence **(A)** and presence of **(B)** DA.

At the starting point, the red-emissive hPEI-Au NCs were synthesized based on the previous report. As indicated in Figure 2A, the fluorescence excitation and emission maxima were centered at 280 and 595 nm, respectively. In addition, the fluorescence excitation and emission spectra were consistent with the reported work (Supplementary Figure S1) (Yang et al., 2020a). These results indicate that red-emissive hPEI-Au NCs were successfully produced. To investigate the interaction between hPEI-Au NCs and DA, the 3D fluorescence emission spectra of hPEI-Au NCs solution with the addition of DA were obtained. As displayed in Figures 2A,B, new emissive species with excitation/emission maxima located at 380/520 nm appeared, while the corresponding emission intensity of hPEI-Au NCs decreased, indicating DA can interact with hPEI-Au NCs and regulate the fluorescence behavior. The newly generated fluorescent species as well as the weakened emission of hPEI-Au NCs make hPEI-Au NCs possible for ratiometric DA sensing in aqueous media and urine.

### Mechanism of DA-Induced Ratiometric Fluorescence Variation

**FIGURE 3:**
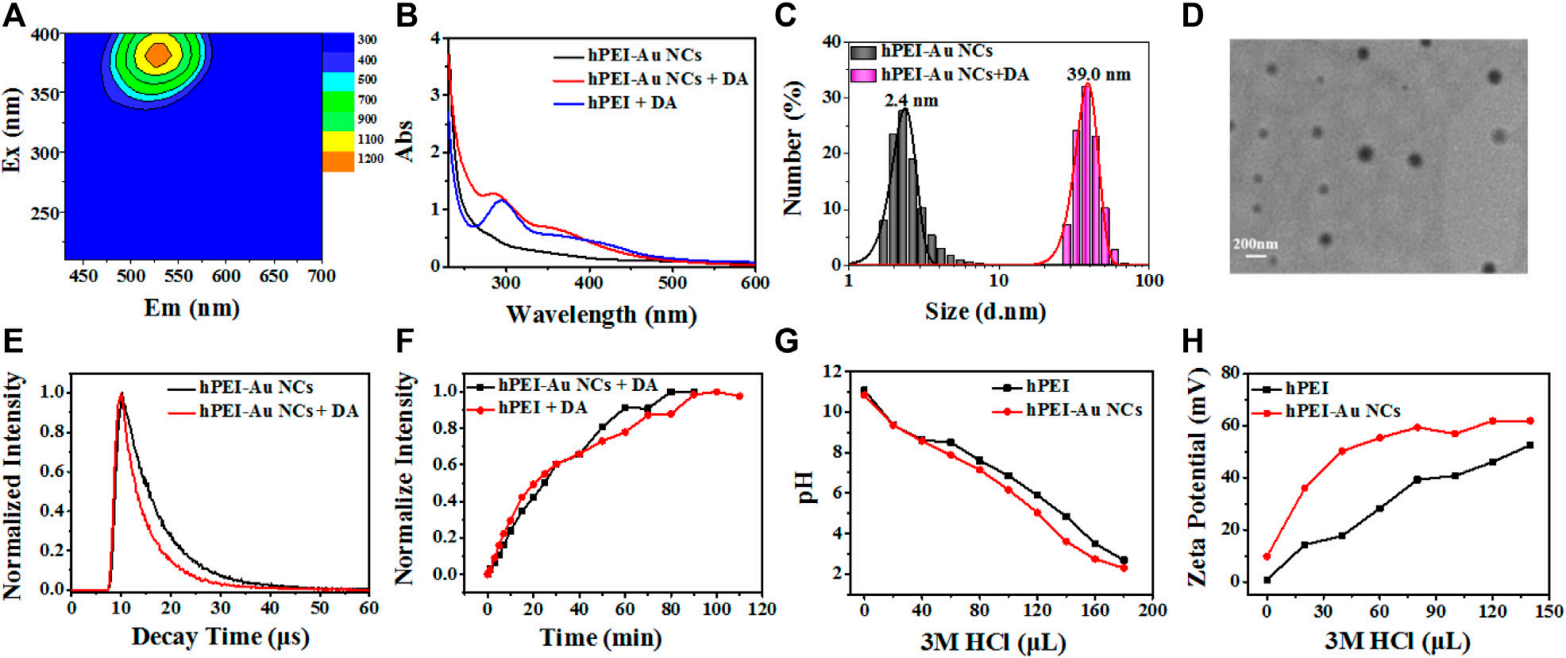
**(A)** 3D fluorescence emission spectra of hPEI solution after adding DA. **(B)** UV–vis absorption spectra of hPEI-Au NCs (black line), hPEI-Au NCs-DA mixture (red line), and hPEI-DA mixture (blue line). **(C)** Hydrodynamic diameter of hPEI-Au NCs without (gray histogram) and with (purple histogram) the addition of DA. **(D)** TEM image of hPEI-Au NCs-DA mixture. **(E)** Time-resolved fluorescence emission spectra of hPEI-Au NCs in the absence (black line) and presence (red line) of DA. **(F)** Time-dependent fluorescence variation of I_520_ of hPEI-Au NCs-DA mixture (black line) and hPEI-DA mixture (red line). **(G)** pH variation of hPEI (black line) and hPEI-Au NCs (red line) upon adding HCl. **(H)** Surface charge variation of hPEI (black line) and hPEI-Au NCs (red line) upon adding HCl.

It is well-known that hPEI could induce the self-polymerization of DA, which forms fluorescent indole intermediate and finally polymeric DA nanoparticles (Liu et al., 2015). To understand the generation of new fluorescent species, 3D fluorescence emission spectra of hPEI solution after adding DA were acquired. As shown in Figure 3A, fluorescent components with maximum excitation and emission wavelengths of 380 and 520 nm appeared, respectively, which is comparable to the product of DA/hPEI-Au NCs mixture, suggesting that the newly produced species are polymeric DA nanoparticles (Sun et al., 2019). After adding DA, a new absorption peak around 380 nm appeared, which is similar to the absorption spectra of polymeric DA nanoparticles (Figure 3B). The similar absorption profiles also demonstrated the generation of polymeric DA nanoparticles. It was seen that the hydrodynamic diameter of hPEI-Au NCs solution showed a dramatic increase after the addition of DA (Figure 3C), from 2.4 to 39.0 nm, indicating the formation of large nanoparticles. According to the TEM image of hPEI-Au NCs/DA mixture, nanoparticles with a size around 90 nm were observed (Figure 3D), further proving the formation of polymeric DA nanoparticles (Sun et al., 2019). According to the TEM, hPEI-Au NCs were not observed nearby polymeric DA nanoparticles. In addition, hPEI is involved in the DA self-polymerization process. Thus, a possible reason is that hPEI-Au NCs were embedded into polymeric DA nanoparticles during the self-polymerization process, as indicated in Figure 1. Moreover, it can be seen from Supplementary Figure S2 that the polymeric DA nanoparticles coated with Au NCs are much larger than the polymeric DA nanoparticles induced by hPEI alone, which also proves our conjecture. As mentioned earlier, the introduction of DA also led to the decrease in red emission of hPEI-Au NCs. To gain insights into the DA-induced fluorescence inhibition mechanism, the time-resolved fluorescence spectra of hPEI-Au NCs without and with the addition of DA were collected. It was seen that the fluorescence lifetime curve of hPEI-Au NCs showed an obvious decrease after reaction with DA (Figure 3E), indicating the alternation of the emission pathway of Au NCs (Lu et al., 2020). Through three components simulation (Supplementary Table S1), the fluorescence lifetime of hPEI-Au NCs decreased from 5.71 to 3.19 µs. As reported in Chang’s work, the fluorescence of thiolate-capped Au NCs originates from ligand-to-metal–metal charge transfer (S→Au ^**…**^ Au, LMMCT), and the rotation of thiolate ligands easily affect the LMMCT pathway and decreases the lifetime (Shiang et al., 2011). Such a phenomenon has also been observed by Zheng and Xie et al. (Sun et al., 2016; Wu et al., 2020). In this case, the formation of polymeric DA nanoparticles leads to the conformation change. However, the emission of Au NCs is largely decided by the MUA ligand and subsequent LMMCT, which is related to the steric structure. The self-polymerization induced conformation change not only alters the steric structure but also regulates the LMMT efficiency. On the basis of our previous work (Zhou et al., 2022), hPEI also contributed to the fluorescence. The conformation change of hPEI may also weaken the interaction and diminish the red fluorescence. As a consequence, the red fluorescence from Au NCs decreased as the DA concentration increased.

It is usually accepted that the reactivity of organic molecules on the nanostructure surface usually exhibits a slight decrease due to the steric effect. To understand whether the hPEI-mediated DA self-polymerization reaction is restricted by Au NCs, the time-dependent fluorescence emission of hPEI and hPEI-Au NCs solutions upon adding DA were investigated. To rule out the concentration-related fluorescence differences, the concentrations of hPEI in both solutions were set to the same. As illustrated in Supplementary Figure S3, the green emission of polymeric DA nanoparticles around 520 nm in both systems gradually increased with the increasing reaction time, and the fluorescence intensity did not show a large difference, indicating the Au NCs encapsulation has no suppression toward DA self-polymerization. It should be noticed that the relative green fluorescence intensity (I_520_) in hPEI-Au NCs solution grew faster than that of hPEI solution (Figure 3F). DA self-polymerization reaction easily occurs under alkaline conditions, and the electron density/nucleophilicity of hPEI plays an important role to enhance the polymerization reaction. In view of these factors, we hypothesized that the accelerated reaction rate may be attributed to the enhanced electron density of the hPEI layer. It is well-known that hPEI can act as a strong proton sponge, which adsorbs abundant protons to primary and secondary amine groups (Wojnilowicz et al., 2019). The strong proton sponge effect makes hPEI a promising candidate for gene/drug delivery. To ensure stable Au NCs encapsulation, strong binding affinity between the amine group in hPEI and the carboxylic group of MUA may exist. This interaction may hinder the adsorption of protons and enhance the electron density of hPEI; as a result, the DA polymerization reaction is boosted. To reveal this assumption, the proton sponge effects of hPEI and hPEI-Au NCs were tested by adding HCl. As shown in Figure 3G, the pH decrease rate of hPEI-Au NCs upon adding HCl was faster than that of hPEI, indicating the decrement of the proton sponge effect after Au NCs encapsulation (Richard et al., 2013). In addition, the surface potential increase rate of hPEI-Au NCs upon adding HCl was also higher than that of hPEI (Figure 3H), further demonstrating the Au NCs encapsulation-induced decrease of the proton sponge effect. Moreover, the slightly increased surface potential may also facilitate the approaching of DA. Thus, Au NCs encapsulation would assist the DA self-polymerization reaction by reducing the proton sponge effect. Taken together, hPEI-assisted DA polymerization not only generates polymeric DA nanoparticles with green emission but also suppresses the red emission of Au NCs by changing the LMMCT pathway, which endows ratiometric and sensitive fluorescence response toward DA.

### DA Sensing in Aqueous Media

Since the introduction of DA into hPEI-Au NCs solution caused dramatic fluorescence variation, such a response might be able to be utilized for fluorimetric DA detection by using hPEI-Au NCs as the optical reporters. As mentioned in our previous report, the self-polymerization reaction of DA in hPEI solution could be affected by several parameters, including pH, solution temperature, and reaction time. To achieve sensitive DA detection, the sensing condition of these factors was optimized. As shown in Supplementary Figure S4A, the formation of polymeric DA nanoparticles was related to solution pH and preferred at pH 10–11. Interestingly, the fluorescence intensity ratio (I_520_/I_595_) of hPEI-Au NCs solution after adding DA was also proportional to pH values (Supplementary Figure S4B). It should be noticed that the pH of hPEI-Au NCs solution was close to 11, thus the following experiments were conducted without further pH adjustment. With Au NCs encapsulation, the temperature-dependent intensity ratio (I_520_/I_595_) showed sine function-like curve (Supplementary Figure S5), which is different from previous studies. It was seen that the red emission from Au NCs displayed a visible decrease under high temperature; the enhanced intensity ratio (I_520_/I_595_) at high temperature should be assigned to the change of Au NCs but not the generation of polymeric DA nanoparticles. As a result, 30°C was chosen as the optimal reaction temperature. In addition, we optimized the reaction time at this temperature. According to the previous works (Du et al., 2014; Li et al., 2021), UV light irradiation can induce the release of reactive oxygen species and thus promote the oxidative polymerization of DA. Therefore, the reaction kinetics was conducted in the absence and presence of UV light radiation (365 nm). As manifested in Supplementary Figure S6, conventional self-polymerization was completed after 50 min reaction, while it became 20 min after UV light radiation, and thus, 20 min reaction window with 365 nm UV light irradiation.

**FIGURE 4:**
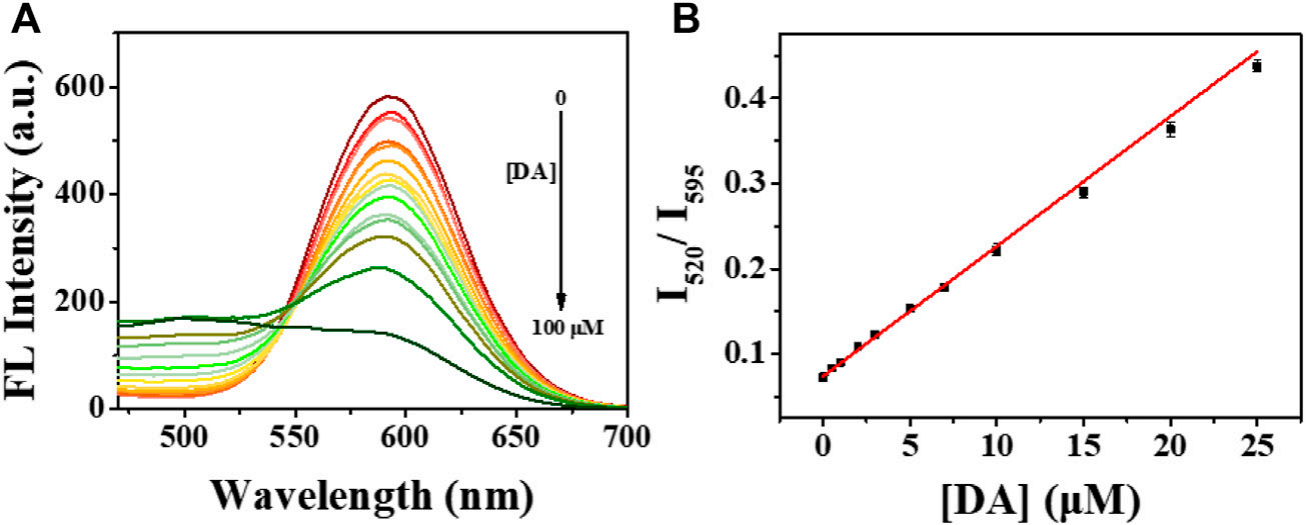
**(A)** Fluorescence emission spectra of hPEI-Au NCs solution upon adding DA with various concentrations. **(B)** Plots of fluorescence intensity ratio (I_520_/I_595_) of hPEI-Au NCs solution vs. DA concentrations.

For conducting ratiometric DA sensing, the sensitivity evaluation was first investigated. After the addition of DA with various concentrations, the fluorescence emission spectra of hPEI-Au NCs solution were obtained. As shown in Figure 4A, the red fluorescence intensity (595 nm) of Au NCs gradually decreased with the increasing DA concentration while without obvious change in the spectral shape. The maintained emission profile indicates the destruction of Au NCs rather than the formation of a new Au NC component. In contrast, green fluorescence steadily increased, yielding an increased fluorescence intensity ratio (I_520_/I_595_). As manifested in Figure 4B, the plots of the intensity ratio (I_520_/I_595_) showed a linear response vs. DA concentration range from 0 to 25 µM. The fluorescence intensity ratio (I_520_/I_595_) can be expressed with a linear equation: y = 0.07 + K [Q] (*R* ^2^ = 0.997), where y is the I_520_/I_595_ value, K is the corresponding fluorescence response constant, and [Q] is the concentration of DA. Through the linear regression of the plots, K was calculated to be 1.5 × 10^4^ M^−1^. To further understand the stability of DA analysis, fifteen repeated measurements of hPEI-Au NCs upon adding certain DA were conducted. As illustrated in Supplementary Figure S7, the fluorescence intensity ratio (I_520_/I_595_) showed only slight variation and a low relative standard deviation (RSD, 1.2%) value, indicating the good reproducibility of the hPEI-Au NC nanoprobe. In addition, four hPEI-Au NC nanoprobes from different batches also displayed small RSD values (2.6%, Supplementary Figure S8), further suggesting the high reproducibility. As a result, the hPEI-Au NC nanoprobe is reproducible for ratiometric DA sensing. Moreover, the LOD toward DA by spectroscopic analysis was determined to be 10 nM (S/N = 3). This LOD is comparable to many reported methods (Supplementary Table S2) (Yu et al., 2014; Guo et al., 2018; Ling et al., 2018; Wang et al., 2018; Naik et al., 2019; Ding et al., 2020; Huang et al., 2020; Liu et al., 2020; Teng et al., 2020; Yao et al., 2020; Baluchova et al., 2021; Chen et al., 2021; Sun et al., 2021; Amara et al., 2022).

**FIGURE 5:**
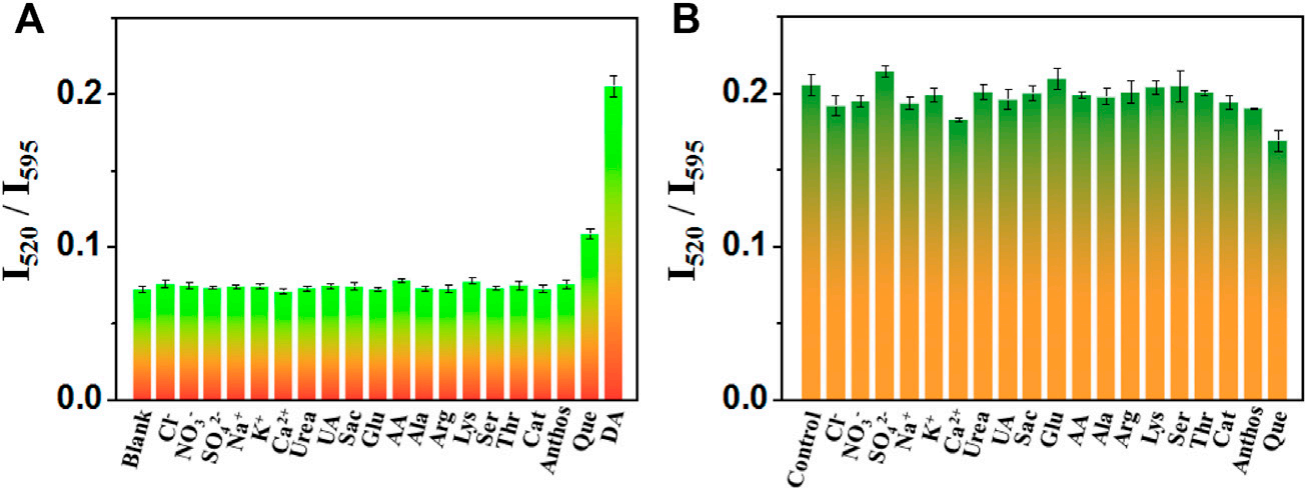
Fluorescence intensity ratio (I_520_/I_595_) of hPEI-Au NCs upon adding DA in the absence **(A)** and presence **(B)** of various interferents.

The selectivity of nanoprobe is also an important character that evaluates its performance. In order to inspect whether the ratiometric fluorescence variation induced by DA is specific, the fluorescence emission spectra of the hPEI-Au NCs nanoprobe after adding possible interferents were recorded. In this work, ions, small molecules, and amino acids, including Cl^−^, NO^3‒^, SO_4_^2‒^, Na^+^, K^+^, Ca^2+^, urea, Sac, Glu, UA, AA, Ala, Arg, Lys, Ser, Thr, Cat, Anthos, and Que were chosen for specificity evaluation. The concentrations of DA and other interferents were 10 µM. As displayed in Figure 5A, none of these interferents, even Cat and Anthos, could generate a conspicuous increment of the intensity ratio (I_520_/I_595_) as DA did, indicating the DA-induced ratiometric change is specific. Although Que showed a slight response, the increased ratio was assigned to its self-florescence around 500 nm. In addition, the change of fluorescence caused by DA is not affected by the addition of various interferents (Figure 5B), which indicates that the proposed nanoprobe has strong interference inhibition. The favorable selectivity is probably attributed to the unique self-polymerization reaction of DA in the presence of hPEI. The hPEI-induced fluorescence response toward these interferents was also recorded. As shown in Supplementary Figure S9, the selectivity and anti-interference were generally the same as the proposed nanoprobe, revealing the unchanged specificity of DA analysis with our system. As a result, for ratiometric DA detection, the hPEI-Au NC nanoprobe is simple, reproducible, and with comparable or better sensing performance in comparison with other reported nanomaterials or fluorophores.

### DA Analysis in Urine Samples

The satisfying selectivity and sensitivity of the hPEI-Au NC nanoprobe suggest a high feasibility of DA detection in real samples. Thus, to further demonstrate the practical application of the proposed nanoprobe, we tried to detect DA in urine samples. In consideration of containing protein and other components, urine samples were centrifuged and filtered to reduce unexpected interference. However, DA in urine samples was not detected, indicating a very low concentration of DA, which is consistent with previous reports. In addition, the detection accuracy was validated by the standard addition method. As summarized in Table 1, the recovery rate was around 100% (91.7–113.5%), proving the practical capability of DA analysis in urine samples. Moreover, the small RSD values (≦ 1.9%, *n* = 3) revealed high reliability for the proposed hPEI-Au NC nanoprobe. In a word, the proposed hPEI-Au NC-based ratiometric platform is capable of DA sensing in complicated biological media.

**TABLE 1:** DA detection in urine samples with the proposed hPEI-Au NCs nanoprobe.

Sample	Spiked DA (μM)	Found (μM)	Recovery (%)	RSD (%,*n* = 3)
Urine	2.00	2.27	113.5	1.1
	3.00	2.75	91.7	1.9
	4.00	3.84	96.0	1.6

## Conclusion

In conclusion, we have explored a ratiometric DA sensing platform using the hPEI-Au NC nanoprobe, which integrates DA self-polymerization and Au NC internal reference. With a self-calibration character, the proposed platform shows high accuracy and sensitivity. In addition, other interferents including ions, small molecules, and amino acids have no visible influence due to the hPEI-mediated specific self-polymerization reaction of DA. By using the hPEI-Au NC nanoprobe, rapid DA detection with an LOD of 10 nM is achieved under the optimized condition. In addition, the practical application is verified by the accurate DA analysis in urine samples. Our study not only develops a ratiometric neurotransmitter sensing system but also demonstrates the exploration of selective nanosensor by involving specific ligand-target reactions. Thus, new protocols for the design of versatile nanosensors for the rapid and selective detection of bioanalytes and other targets by integrating functional nanomaterials and unique chemical reactions are now possible in life science or environment-related fields.

## Data Availability

The original contributions presented in the study are included in the article/Supplementary Material; further inquiries can be directed to the corresponding authors.
