# Supplementary material for: Gold Nanocluster-Encapsulated Hyperbranched Polyethyleneimine for Selective and Ratiometric Dopamine Analyses by Enhanced Self-Polymerization
Source: Front Chem. 2022 Jul 8;10:928607. doi: 10.3389/fchem.2022.928607 (PMC9307107; doi:10.3389/fchem.2022.928607)
Supplement: Supplementary file 1 [file DataSheet1.doc]

Supporting Information for:

Gold nanocluster-encapsulated hyperbranched polyethyleneimine for selective and ratiometric dopamine analysis by enhanced self-polymerization

Jing Zhang,^a^ Ying Liu,^a^ Yang Liu,^a^ Wencai Liu,^a^ Fengniu Lu,^b^ Zhiqin Yuan*^a,c^ and Chao Lu*^a,d^

*^a^ State Key Laboratory of Chemical Resource Engineering, College of Chemistry, Beijing University of Chemical Technology, Beijing 100029, China*

*^b^ Department of Chemistry and Chemical Engineering, Beijing Institute of Technology, Beijing, 100081, China*

*^c^ Beijing Key Laboratory of Plant Resources Research and Development, Beijing Technology and Business University, Beijing 100048, China*

*^d^ Green Catalysis Center, College of Chemistry, Zhengzhou University, Zhengzhou 450001, China*


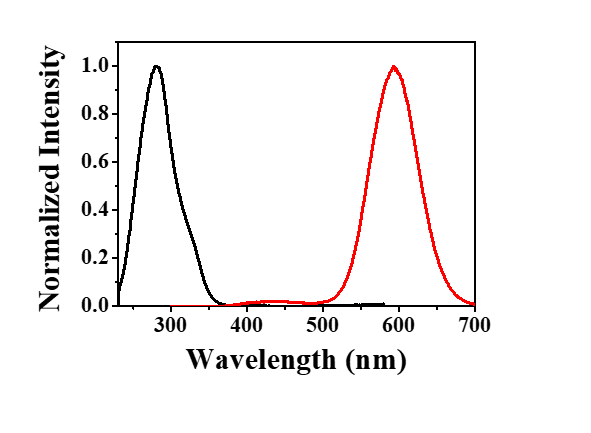


**Figure S1.** Fluorescence excitation (black line) and emission (red line) spectra of hPEI-Au NCs.


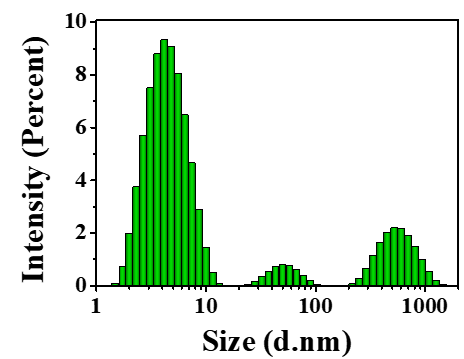


**Figure S2.** Hydrodynamic diameter of polydopamine nanoparticles induced by hPEI.


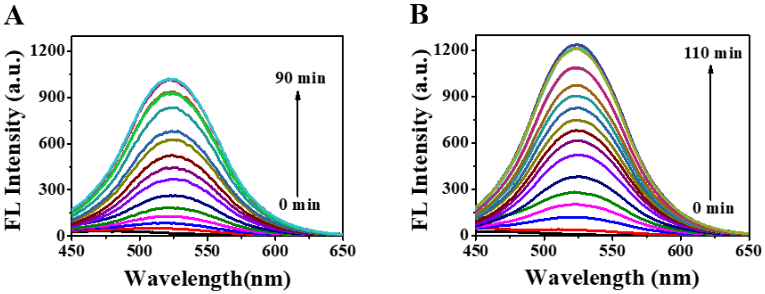


**Figure S3.** Time-dependent fluorescence emission spectra of hPEI-Au NCs/DA mixture (A) and hPEI/DA mixture (B) upon excitation at 380 nm.


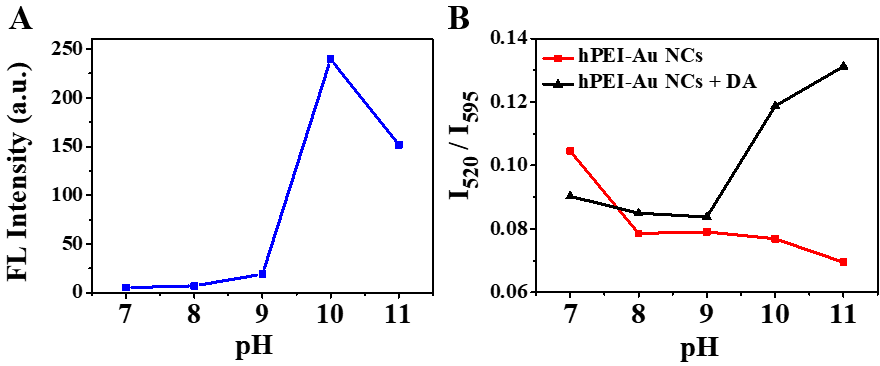


**Figure S4.** (A) The green fluorescence intensity (I_520_) of hPEI/DA mixture *versus* solution pH from 7 to 11. (B) The fluorescence intensity ratio (I_520_/I_595_) of hPEI-Au NCs (red line) and hPEI-Au NCs/DA mixture (black line) *versus* solution pH from 7 to 11.


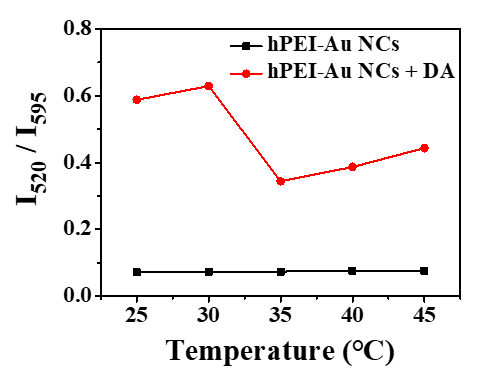


**Figure S5.** The fluorescence intensity ratio (I_520_/I_595_) of hPEI-Au NCs (black line) and hPEI-Au NCs/DA mixture (red line) *versus* reaction temperature.


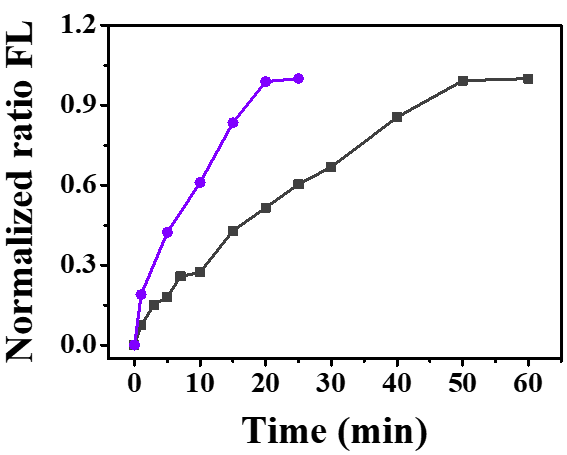


**Figure S6.** Time-dependent fluorescence intensity ratio (I_520_/I_595_) variation of hPEI-Au NCs-DA mixture in the presence (purple line) and absence (gray line) of UV (365 nm).


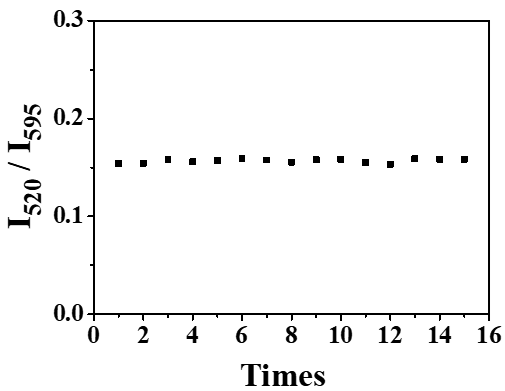


**Figure S7.** Fluorescence intensity ratio (I_520_/I_595_) of hPEI-Au NCs in the presence of DA with 15 repeated measurements.


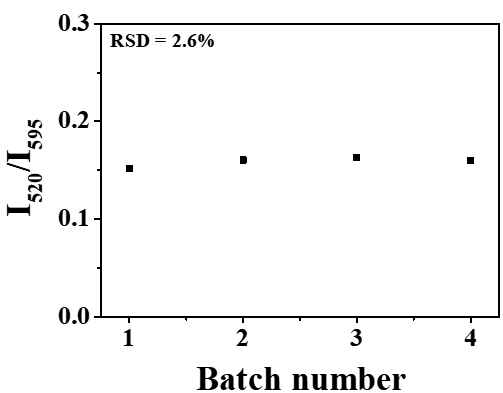


**Figure S8.** Fluorescence intensity ratio (I_520_/I_595_) of four hPEI-Au NC nanoprobes in the presence of DA .


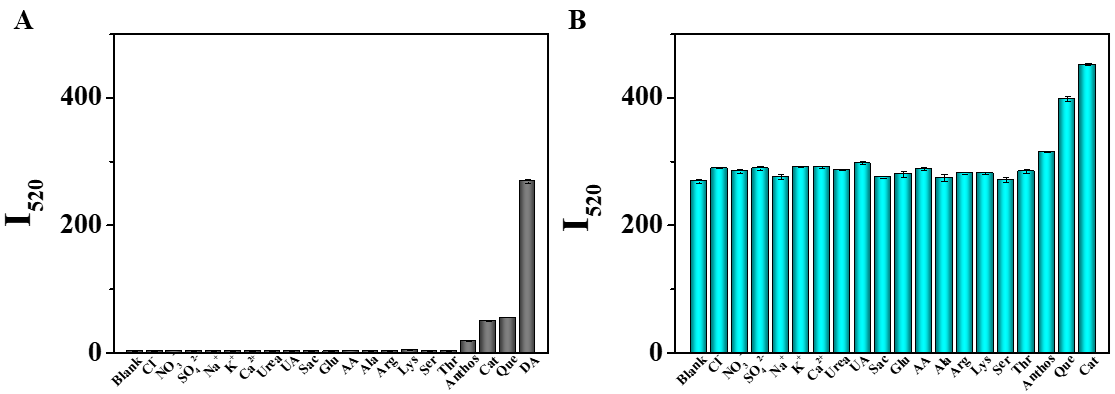


**Figure S9.** Fluorescence intensity (I_520_) of hPEI upon adding DA in the absence (A) and presence (B) of various interferents.

**Table S1. The multiexponential decay fitting results of fluorescence lifetimes of hPEI-Au NCs upon adding DA.**

| Added DA (μM) | τ_ave_ (μs) | τ_1_ (μs) | τ_2_ (μs) | τ_3_ (μs) |
| --- | --- | --- | --- | --- |
| 0 | 5.71 | 1.00 (16.79%) | 6.40 (74.81%) | 9.00 (8.40%) |
| 100 | 3.19 | 1.00 (47.85%) | 5.00 (50.00%) | 10.00 (2.15%) |

**Table S2. Comparison of this work with some established DA detection methods.**

| **No.** | **Materials** | **Strategy** | **Linear range**  **(μM)** | **LOD**  **(μM)** | **Ref.** |
| --- | --- | --- | --- | --- | --- |
| 1 | N-C dots | Turn-off luminescence | 10.55 – 105.5 | 10.39 | Naik et al., 2019 |
| 2 | Pdots@AMP-Cu | Turn-off luminescence | 10 - 400 | 4.00 | Huang et al., 2020 |
| 3 | [C_12_ImCONH_2_]Br/Eu-POM | Turn-off luminescence | 0 - 100 | 0.10 | Guo et al., 2018 |
| 4 | Cys-AIZS QDs | Turn-off luminescence | 15 - 120 | 0.65 | Liu et al., 2020 |
| 5 | IL-MXene/GPE Electrode | Electrochemical | 10 - 2000 | 0.702 | Amara et al., 2022 |
| 6 | PANI/GCE Electrode | Electrochemical | 0.5 - 300 | 0.118 | Chen et al., 2021 |
| 7 | NG/PEDOT hybrids Electrode | Electrochemical | 0.2 - 90 | 0.054 | Teng et al., 2020 |
| 8 | Au nanospheres Electrode | Electrochemical | 5 - 1000 | 0.8 | Yao et al., 2020 |
| 9 | Porous H-BDD Electrode | Electrochemical | 0.7 - 30 | 0.2 | Baluchova., 2021 |
| 10 | ENA/ITO Electrode | Electrochemiluminescence | 0.25 - 1.12 | 0.23 | Ding et al., 2020 |
| 11 | ZnIn_2_S_4_/CdS | Photoeletrochemical | 0.3 - 300 | 0.10 | Wang et al., 2018 |
| 12 | BA-Tb-MOG | Ratiometric fluorescence | 1 - 30 | 0.08 | Sun et al., 2021 |
| 13 | NaGdF_4_:Tb NPs | Turn-off luminescence | 0 - 20 | 0.03 | Ling et al., 2018 |
| 14 | ErGO Electrode | Electrochemical | 0.1 - 10 | 0.10 | Yu et al., 2014 |
| 15 | hPEI-Au NCs | Ratiometric fluorescence | 0 - 25 | 0.01 | This work |
