# Supplementary material for: Gold Nanocluster-Encapsulated Hyperbranched Polyethyleneimine for Selective and Ratiometric Dopamine Analyses by Enhanced Self-Polymerization
Source: Front Chem. 2022 Jul 8;10:928607. doi: 10.3389/fchem.2022.928607 (PMC9307107; doi:10.3389/fchem.2022.928607)
Supplement: Supplementary file 2 [file DataSheet2.docx]

**Graphical abstract**


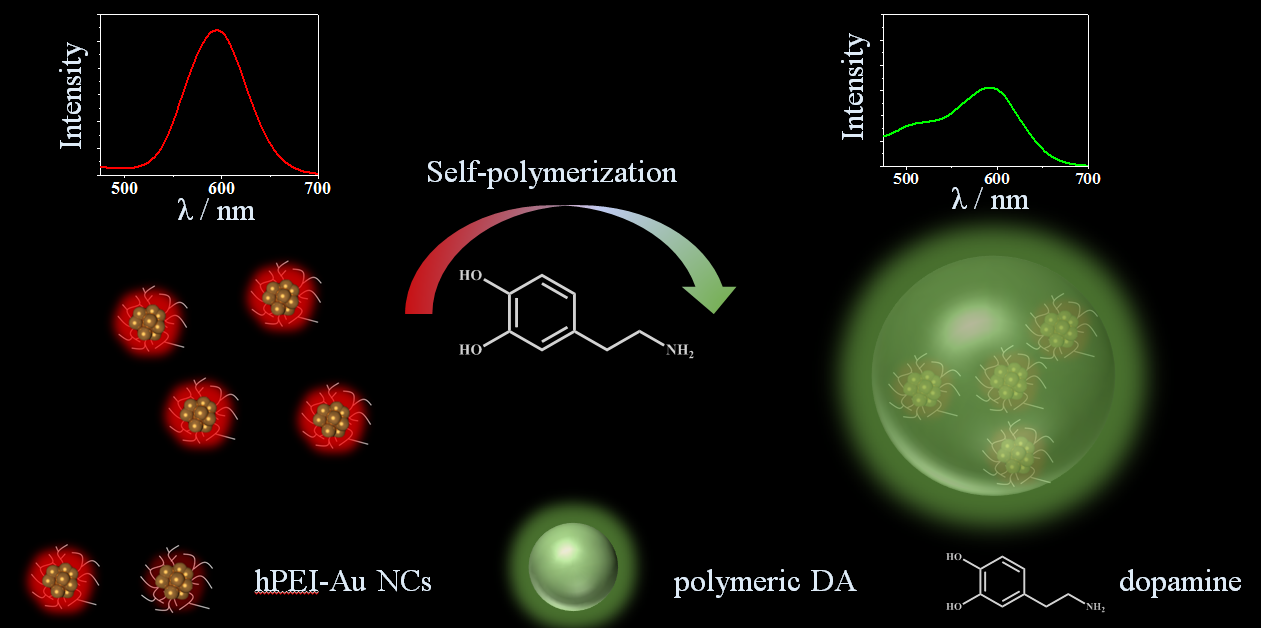


Selective and ratiometric dopamine detection is realized by gold nanocluster-encapsulated hyperbranched polyethyleneimine enhanced self-polymerization.
